# Use of transcriptome sequencing to understand the pistillate flowering in hickory (*Carya cathayensis* Sarg.)

**DOI:** 10.1186/1471-2164-14-691

**Published:** 2013-10-10

**Authors:** You-Jun Huang, Li-Li Liu, Jian-Qin Huang, Zheng-Jia Wang, Fang-Fang Chen, Qi-Xiang Zhang, Bing-Song Zheng, Ming Chen

**Affiliations:** 1 The Nurturing Station for the State Key Laboratory of Subtropical Silviculture, Zhejiang A&F University, Lin’an, Zhejiang 311300, China; 2 Department of Bioinformatics, State Key Laboratory of Plant Physiology and Biochemistry, College of life Science, Zhejiang University, Hangzhou, Zhejiang 310058, China

**Keywords:** Co-expression network, *Carya cathayensis* Sarg, Floral development, Seasonal flowering, Hickory, High-throughput data analysis

## Abstract

**Background:**

Different from herbaceous plants, the woody plants undergo a long-period vegetative stage to achieve floral transition. They then turn into seasonal plants, flowering annually. In this study, a preliminary model of gene regulations for seasonal pistillate flowering in hickory (*Carya cathayensis*) was proposed. The genome-wide dynamic transcriptome was characterized via the joint-approach of RNA sequencing and microarray analysis.

**Results:**

Differential transcript abundance analysis uncovered the dynamic transcript abundance patterns of flowering correlated genes and their major functions based on Gene Ontology (GO) analysis. To explore pistillate flowering mechanism in hickory, a comprehensive flowering gene regulatory network based on *Arabidopsis thaliana* was constructed by additional literature mining. A total of 114 putative flowering or floral genes including 31 with differential transcript abundance were identified in hickory. The locations, functions and dynamic transcript abundances were analyzed in the gene regulatory networks. A genome-wide co-expression network for the putative flowering or floral genes shows three flowering regulatory modules corresponding to response to light abiotic stimulus, cold stress, and reproductive development process, respectively. Totally 27 potential flowering or floral genes were recruited which are meaningful to understand the hickory specific seasonal flowering mechanism better.

**Conclusions:**

Flowering event of pistillate flower bud in hickory is triggered by several pathways synchronously including the photoperiod, autonomous, vernalization, gibberellin, and sucrose pathway. Totally 27 potential flowering or floral genes were recruited from the genome-wide co-expression network function module analysis. Moreover, the analysis provides a potential *FLC*-like gene based vernalization pathway and an 'AC’ model for pistillate flower development in hickory. This work provides an available framework for pistillate flower development in hickory, which is significant for insight into regulation of flowering and floral development of woody plants.

## Background

Flowering is a vital event in plant growth and development through which alternation of generations from vegetative growth to reproductive growth is accomplished [1]. It is an intricate biological and morphological process which is regulated by a large number of genes. Most studies of flowering mechanisms have focused on herbal model plants (e.g. *Arabidopsis thaliana* and *Antirrhinum majus*) [2-9]. Five pathways in flowering process have been designated i.e., the photoperiod pathway, the autonomous pathway, the vernalization pathway, the gibberellin pathway and the sucrose pathway [10,11]. Each route responds to endogenous or environmental cues relatively independently but acts jointly during late stages and intertwines a comprehensive network via floral integrators such as *Flowering Locus T* (*FT*), *SUPPRESSOR OF OVEREXPRESSION OF CONSTANS 1* (*SOC1*) and *AGAMOUS-LIKE 24* (*AGL24*). Subsequently, these floral integrators trigger floral meristem identifying genes *LEAFY* (*LFY*) and *APETALA1* (*AP1*) and promote flowering [12,13]. Recently, comprehensive insights of first flowering and seasonal flowering were obtained from studies in perennial plants e.g. *Arabis alpine*. The differences in histone modifications at Flowering Locus C (FLC) and PEP1 (the orthologue of the *A. thaliana* gene FLC) in *A. thaliana* and *A. alpine* may be one of the mechanisms by which these alterations in gene expression patterns occur, thereby allowing diversification of rapidly evolving traits such as life history characters [14]. TERMINAL FLOWER 1 (TFL1) in *A. alpine* (AaTFL1) blocks flowering by setting a threshold for a flowering pathway and prevents LEAFY in *A. alpine* expression when young plants are exposed to vernalization. Vernalization of the older *A. alpine* plants reduces expression of floral repressor PEP1 and activates AaSOC1 and AaLFY, then promotes flowering [15]. This developmental transition in perennials is probably more complex than in other plants and the molecular mechanisms are less well understood. In addition, once perennials become adult and capable of reproduction they still keep some meristems in the vegetative state that contribute to their polycarpic growth habit. Juvenility and polycarpy, although considered as two different processes in perennials, might be related [16].

**Figure 1:**
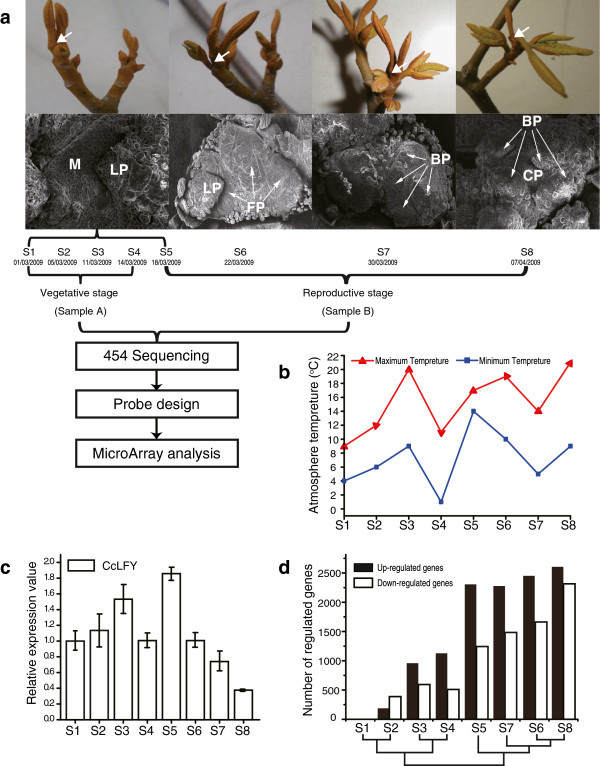
**Experimental design. (a)** Floral developing process by morphological and ultrastructure (scanning electron microscopy, SEM) observation. White arrows in short-pod-branches indicate apical meristem where pistillate floral buds initiate. Eight samples in timing order, namely S1, S2, S3, S4, S5, S6, S7, S8. Mixed pistillate flower buds of S1-S5 and S6-S8 namely SampleA and SampleB, respectively. M, meristem. LP, leaf primordia. FP, flower primordia. BP, bract primordia. CP, carpel primordia. SampleA and SampleB were carried out to do 454 transcriptome sequencing, respectively. Sequenced reads were assembled to contigs. Microarrays were designed which probes came from all of contigs. Microarrays are hybridized with cDNA at eight time point of S1-S8, respectively. **(b)** Ambient temperature record of at each stage. **(c)***CcLFY* expression by QRT-PCR as a reference to explore the turning point of floral determination at the molecular level. **(d)** Number of genes differential expression over flower development.

Woody plants need a long vegetative period to achieve transition to the reproductive stage [17,18]. After this transition, trees begin to form flower buds in the spring of each growing season [19]. Each seasonal flowering period is interrupted by a long vegetative period [20]. As a famous nut tree in China, hickory (*Carya cathayensis* Sarg.) is similar to the model woody plant poplar in several biological aspects. Both species are woody, deciduous and catkin-bearing plants with a long juvenile stage. Their pistillate flowers are naked without perianth. On the aspect of biological characteristics, the pistillate flower in hickory initiates from a terminal bud which grows in short pod-branches as a young hickory tree lives at a reproductive age. Generally, the pistillate flower bud differentiates morphologically from late March each year after hibernation release. Previous research suggests that the morphological turning point from vegetative to productive stage emerges in late March as male inflorescence buds are dehiscent (Figure 1a; [21]). In advance, *CcLFY* (GenBank accession number: DQ989226), which is a homolog of *LFY* in hickory, was applied as a landmark to explore the turning point of flower-bud determination at molecular level.

However, knowledge about the molecular genetics of flowering time results from studies in *A. thaliana*[10,18]. It is still poorly understood about which genetic factors control first-time and seasonal flowering, about how many pathways take part in the process in poplar [22]. In recent years, a few researchers set out to study the molecular mechanism of first-time and seasonal flowering and made some process. It is reported that *FT* duplication coordinates reproductive and vegetative growth in poplar [19]. CONSTANS (CO) and FT are involved in the initiation of photoperiod-dependent dormancy [23]. The CO/FT regulatory module controls timing of flowering and seasonal growth cessation in trees [24].

Taken together, *A. thaliana* was chosen as a contradistinctive material to study the flowering network of pistillate flower development in hickory. In this paper, the joint-approach of RNA sequencing and microarray analysis was employed to discover new flowering or floral genes and to show the regulation of the seasonal flowering mechanism in hickory. Microarray is considered a 'close’ platform because only the genes spotted on the arrays can be analyzed. In contrast, the 'open’ platform of 454-sequencing of cDNAs can give transcript profiles without prior knowledge of the genes to be identified and thus enable the discovery of new expressed genes [25]. As a result, ten thousands of abundant transcripts during hickory flower development were identified, and the kinetics of the patterns in pistillate flower ontogeny was determined. Even more momentously, a gene seasonal flowering co-expression network in hickory was constructed. Understanding the process of flowering or floral development in hickory helps to understand the flowering mechanisms of woody plants in general.

## Results

### Characterization of transcriptome dynamics associated with hickory flower ontogeny

#### 454 sequencing data

To determine the hickory transcriptome during flower development, two mRNA libraries (SampleA and SampleB) were designed for RNA-seq. More than 800,000 reads produced from 454 sequencing were assembled into 25,339 contigs for SampleA and 26,935 for SampleB, respectively. After blast analysis between SampleA and SampleB, 4,951 SampleA specific contigs and 5,887 SampleB specific contigs were identified. A large number of common contigs with e value ≤ 1e-10 were obtained as well, including 20,388 from SampleA and 21,048 from SampleB (Additional file 1: Table S1). Thereafter, probes were designed based on assembled 454 contigs and 109 floral core genes of *A. thaliana*. Microarrays for the time points S1-S8 were hybridized as pistillate flowering transcript abundance profiles.

#### Transcriptome dynamics and function enrichment analysis

The hickory microarray slides were used to investigate the transcript abundance profiles of hickory flowering and floral development during S1-S8 stages. The distribution of the differentially transcribed probe sets over pistillate flowering is illustrated in Figure 1d. The kinetics of the transcripts abundance shows that there are rather few significantly differentially transcribed probe sets in the first four samples, S1-S4, while there is a considerably higher amount of differentially transcribed probe sets in the later samples S5-S8. This is probably due to the fact that the terminal buds in the short pod-branches of hickory are going through a dormant period before entering an active growth stage. In addition, the number of differentially transcribed probe sets at the point of S5 increases strikingly compared to the points before S5. The result suggests that S5 is a turning point from the vegetative to reproductive stage at the molecular level in hickory. This is also in accordance with the quantitative expression of *CcLFY* which peaked at the point of S5 (Figure 1c). It is suggested that S5 stage is the critical point of pistillate flower bud differentiation at the molecular level and occurs at least four days earlier than that at morphological or anatomical level at S6 stage (Figure 1a). The number of down-regulated probe sets is larger than up-regulated genes at the second time point, indicating that the onset of flower development is accompanied by the repression of many genes. The ratio between up- and down-regulated genes shifted subsequently after the second sample, as considerably more genes were activated than repressed after the S2. These cases are similar with the *A. thaliana* transcriptome profile during early flower development [26,27]. The sample clustering as shown in Figure 1d, identifies two major categories. One cluster relates to the stage of flower bud undifferentiation whereas alternately cluster biases the period of flower bud differentiation. In addition, S1 and S2 are highly similar in transcript abundance patterns, with more down- than up-regulated genes in order to maintain bud dormancy (Figure 1d). However, S3 and S4 have more up- than down-regulated genes to prepare for breaking the dormancy and to enter the active growth period. Interestingly, the result indicates that S6 and S8 are clustered to a group rather than S7 and S8. One possible reason is that the minimum temperature suddenly drops from 10°C at S6 to 5°C at S7, however the temperature is almost equal at S6 and S8 (Figure 1b). Lower temperature probably influences the normal metabolism and molecular regulation and consequently decreases the number of differential transcripts.

**Figure 2:**
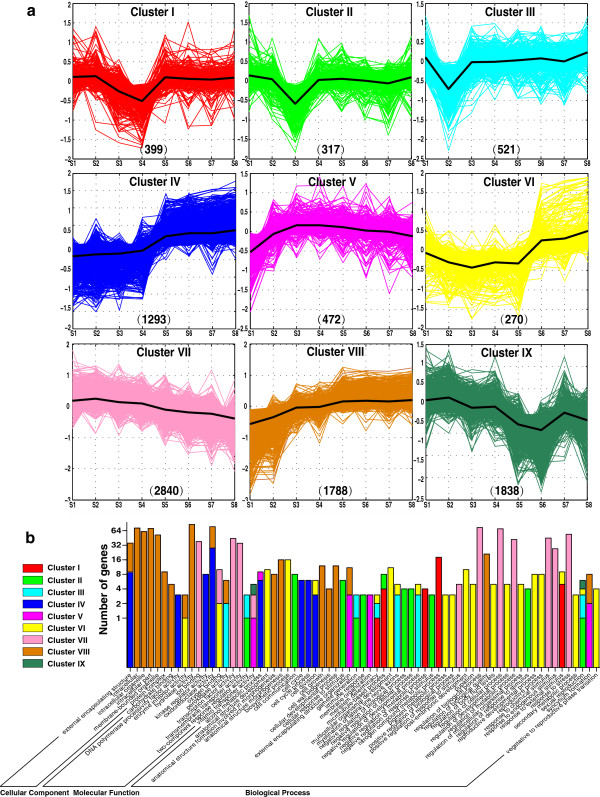
**Dynamic expression pattern of different clusters during flower development and GO function enrichment analysis. (a)** Dynamic expression pattern of different clusters. Transcriptome profiling in pistillate flower buds of hickory shows highly coordinated expression during flower development. Nine major type patterns were identified, which were denoted as different color, respectively. The number of genes for each cluster represents in each panel. **(b)** Functional categorization of genes from different clusters. The color sets for each cluster are in strict accordance with part **(a)**.

Furthermore, to characterize the transcriptome dynamics of flower ontogeny from vegetative to reproductive stage, a total of 8,937 significantly differential transcripts were identified using an arbitrarily fourfold change criterion. K-means clustering of the 8,937 differential transcripts identified nine major types of patterns (Figure 2a). These clusters reflect the general trends and key transitional states during pistillate flowering. Cluster III, II, I and IX comprise genes that were down-regulated at different time points and reached their lowest transcript level at S2, S3, S4 and S6, respectively. The genes in cluster IV and VI were up-regulated at S4 and S5, respectively, and then retained the same transcript level in the later samples. Cluster V kept genes up-regulating in the first samples and then down-regulating after S3. Genes in Cluster VII showed a minor decrease in transcript abundance, while Cluster VIII genes exhibited a minor increase during flower development.

The gene ontology (GO) annotation, corresponding to cellular component, molecular function and biological process, is applied to assign each cluster to statistically enrichment functional categories (Figure 2b). First, probe sets in cellular component category mostly attribute to Cluster VII, and a little to Cluster IV. In detail, cell division patterns are regulated differently at different stages of flowering time and floral development [28]. For instance, XAANTAL1 (XAL1), an upstream regulator of SOC1, FT and LFY, regulates cell proliferation of additionally aerial meristems [29]. Actin-depolymerizing factor (ADF) regulates dually flowering and cell expansion and organ growth [30]. The *FRUITFULL* (*FUL*) gene mediates cell differentiation during *A. thaliana* fruit development [31]. Their homologs in hickory could play similar roles as in *A. thaliana* during flowering.

Moreover, it is reported that some protein complexes in *A. thaliana*, such as DDB1-CUL4 ASSOCIATED FACTOR1 (DCAF1) and DDB1 binding WD40 (DWD) complexes function in photoperiod pathway [32]. The RNA polymerase II associated factor (PAF) and the SWR1 complex (SWR1c) function in autonomous pathway [33,34]. PLANT HOMEO DOMAIN-POLYCOMB REPRESSIVE COMPLEX2 (PHD-PRC2) and DELLA complexes function in vernalization and gibberellin pathway, respectively [35,36]. It is speculated that these protein complexes have some functions during hickory flowering as well.

In the molecular function category, major functions associated with pistillate flowering were identified by GO enrichment analysis (shown in Figure 2b). Enzyme inhibitor, for example, plays vital roles in flowering. For instance, overexpression of a trypsin inhibitor AtKTI1 causes early-flowering in *A. thaliana*[37]. Aminooxy acetic acid (AOA) and L-2-aminooxy-3-phenylpropionoic acid (AOPP) function as phenylalanine ammonia-lyase (PAL) inhibitors inhibit stress-induced flowering [38]. Transcription factors attribute solely to one cluster i.e. cluster VII, which trend down-regulates slowly in entire flowering process. The result implies that most transcription factors acts as negative floral regulators to regulate flowering in hickory. Especially members of MADS domain transcription factors are key floral genes. For example, FLC encodes a MADS domain protein that acts as a repressor of flowering [39]. In addition, other transcription factors such as SBP-box transcription factors, NAC-domain transcription factors, bZIP transcription factors, CCAAT-binding transcription factors, KNOX transcription factors, NF-Y transcription factors, Myb-like transcription factor, zinc finger transcription factors, bHLH transcription factors, GATA-type transcription factors, are essential in flowering.

In the biological process category, vegetative to reproductive phase transition, positive regulation of biological process, regulation of developmental process, regulation of multicellular organismal process, reproductive developmental process, reproductive process attribute to Cluster VI. In detail, putative flowering time genes such as homologs of *COLD, CIRCADIAN RHYTHM, AND RNA BINDING 2* (*CCR2*), *FLOWERING LOCUS D* (*FLD*), *FPA*, *protein arginine methyltransferase 10* (*AtPRMT10*), *with no lysine kinase 8* (*WNK8*), *glucose-1-phosphate adenylyltransferase* (*ADG1*), *CONSTITUTIVE PHOTOMORPHOGENIC 1* (*COP1*), *EARLY FLOWERING 4* (*ELF4*), *MADS AFFECTING FLOWERING 1* (*MAF1*), *methyl‒CpG‒binding domain* (*MBD9*), *cullin4* (*CUL4*) and *CIRCADIAN CLOCK ASSOCIATED 1* (*CCA1*) attribute to vegetative to reproductive phase transition. In the reproductive developmental process, several putative flowering genes such as homologs of *squamosa promoter-binding-like protein 3* (*SPL3*), *AGL24*, *EARLY FLOWERING 8* (*ELF8*), *EMBRYONIC FLOWER 1* (*EMF1*), *MAF1*, *FLD*, *FPA*, *PHYTOCHROME AND FLOWERING TIME 1* (*PFT1*) play important roles particularly in morphological floral transition. In addition, cluster VII shows a minor decrease in transcript abundance mostly involved in positive regulation of response to stimulus, regulation of biological process, response to chemical stimulus, response to endogenous stimulus, response to stress. For example, some putative flowering genes in Cluster VII such as homologs of *CCA1*, *ELF6*, *AGL24*, *RGA-Like 2* (*RGL2*), *SPINDLY* (*SPY*), *CULLIN4* (*CUL4*), *VITAMIN C DEFECTIVE 1* (*VTC1*), *PFT1* belong to GO function of response to endogenous stimulus, while some putative flowering genes such as homologs of *AtSUC3*, *SYD*, *EBS*, *COP1*, *CUL4*, *FVE*, *AGL24*, *SHK1 KINASE BINDING PROTEIN1* (*SKB1*), *MAF2*, *PFT1*, *CCA1*, *CCR2*, *VERNALIZATION INSENSITIVE 3* (*VIN3*), *VTC1* belong to GO function of response to stress. Both categories of flowering genes regulate flowering negatively. Moreover, negative regulation of cellular process, negative regulation of biological process, cellular response to stimulus, regulation of response to stimulus, negative regulation of response to stimulus, gamete generation, attribute to Cluster II. Response to external stimulus attributes mainly to Cluster I, which down-regulates until S4 and subsequently up-regulates after S5, while a little to Cluster VI. Genes involving in meristem determinacy attribute to Cluster I, III and VI and genes involving in sexual reproduction attribute dispersedly to Cluster II, III, VI and IX. The GO annotation analysis provides system-level insights into the pistillate flowering.

### Towards a hickory dynamic flowering network

For identifying the flowering and floral genes in hickory, BLASTN searches for all of the contigs from SampleA and SampleB have been done against a local *A. thaliana* cDNA sequence database. As a result, a total of 84 hickory flowering or floral relative genes were identified, including 21 SampleA specific genes, 31 SampleB specific genes and 32 genes common for both samples (see Additional file 2: Table S7 for the blast results and corresponding sequence information). In addition, 109 flowering or floral core genes of *A. thaliana* consulted from more than 1000 literatures were designed to construct part of the whole probe pool, of which 31 genes have reliable hybridization signal value with hickory. Finally, 114 flowering or floral relative genes in hickory were identified (See Additional file 3: Table S3 for a complete list of the identified flowering or floral relative genes in hickory).

**Figure 3:**
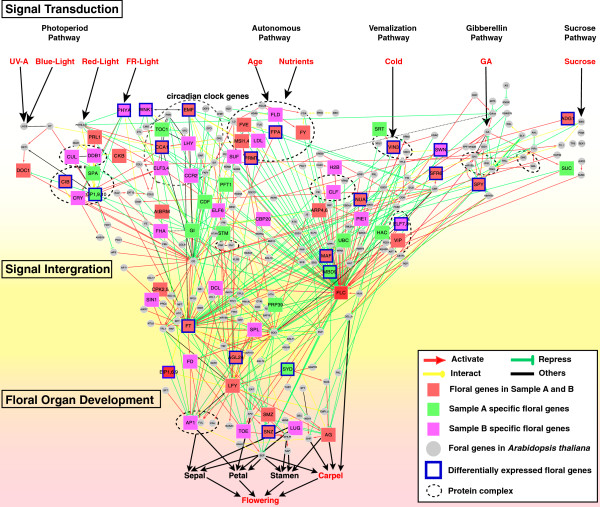
**Flowering network in hickory based on that in Arabidopsis It shows 3 stages latitudinally (signal transduction, signal integration, floral organ development) or 5 pathways (photoperiod, vernalization, autonomous, gibberellic acid (GA), and sucrose pathways) longitudinally to flowering.** Circle in gray presents a flowering or floral gene lies in *A. thaliana* but hasn’t been discovered in hickory. Rectangle in reddle presents a common flowering or floral gene in both SampleA and SampleB. Rectangle in green presents a specific flowering or floral gene in SampleA, while Rectangle in pink presents that in SampleB. Rectangle with blue border presents a differentially expressed flowering or floral gene. Circle in dotted line presents a protein complex.

Due to lack of previous functional studies in hickory, the flowering network in hickory was grounded on foregoing reports of flowering and floral development in *A. thaliana*. A total of 390 genes related to flowering were acquired from the published literatures since 1990s. By exploring their functions and the regulations, a comprehensive flowering network in *A. thaliana* was reconstructed (Figure 3, Additional file 4: Table S2), which showed 3 stages latitudinally (signal transduction, signal integration, floral organ development) and 5 pathways (designated as the photoperiod, the autonomous, the vernalization, the gibberellin, and the sucrose pathways) longitudinally [10,11]. A total of 114 putative flowering or floral genes, including 31 differentially transcribed putative flowering or floral genes were found homologous genes in hickory and mapped to the comprehensive flowering regulatory network shown in Figure 3. These putative flowering or floral genes are distributed in all latitudinal stages and pathways in the network. It is suggested that the flowering event of pistillate flower bud in hickory keeps intricate and involves several pathways synchronously including the photoperiod, the autonomous, the vernalization, the gibberellin, and the sucrose pathways. That is, flowering in hickory is a comprehensive phenomenon with internal and external stimuli, the former includes age, nutrients, endogenous hormones, and the later includes light, day-length, temperature, stress, etc.

#### Differentially transcribed putative flowering or floral core genes

**Figure 4:**
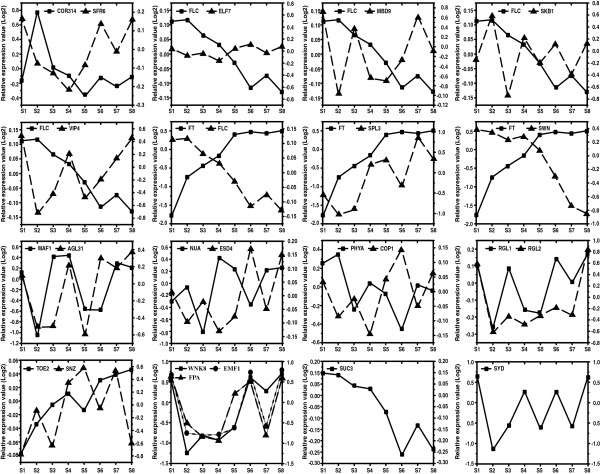
**Transcriptional regulations of differentially expressed genes in flowering and floral development in hickory.** Left Y-axis is appropriate for a gene in solid line, while right Y-axis is for a gene in dotted line.

A total of 31 differentially transcribed putative flowering or floral core genes were captured in hickory (Figure 3, Additional file 3: Table S3). They regulate or are regulated by other putative flowering or floral genes and play vital roles in hickory flowering (Figure 4). Of them, 3 genes, homologs of *PHYTOCHROME A* (*PHYA*), *COP1* and *CRYPTOCHROME-INTERACTING BASIC-HELIX-LOOP-HELIXs* (*CIBs*), are involved in the photoperiod pathway in *A. thaliana*. *PHYA-like* shows a decrease in transcript abundance during the early stages in hickory. It reaches lowest transcript levels at S6 and then stays at relatively low levels in the subsequent stages of hickory floral development. Compared to the *PHYA-like*, the transcript abundance of *COP1-like* fluctuates narrowly in hickory. *CIB5-like* attributes to Cluster II, whereas *CRYPTOCHROME 2* (*CRY2*)*-like* is rather constantly transcribed throughout S1-S8.

Several flowering genes *WNK1*, *WNK8* (*EIP1*), *EMF1*, *PSEUDO-RESPONSE REGULATOR 7* (*PRR7*) and *CCA1* act as circadian clock genes in photoperiod pathway in *A. thaliana*. Their homologs are differentially transcribed in hickory. In detail, the *WNK8-like* and *EMF1*-like genes have similar transcript abundance patterns during flowering in hickory. Both genes show a decrease in transcription level from S1 to S2, remain at a low level from S3 to S5 and then become up-regulated at the later stages. The transcript abundance of *SKB1-like* fluctuates while its downstream gene *CcFLC* (GenBank: JQ829074.1) goes down during flowering. The result shows that their transcript abundance patterns are different from each other because *FLC* is a key target of many upstream genes including *SKB1*, which is a weak suppressor and whose minor effect also indicates further redundancies in the repression of *FLC* in *A. thaliana*[40]. A VIN3 relative is suggested to negatively regulate CcFLC during flowering in hickory based on flowering regulatory network in *A. thaliana* and the transcript abundance pattern comparison with *A. thaliana*. It is reported that *ELF7* (*VIP2*) is required for a high level of *FLC* expression in *A. thaliana*[41]. However in hickory, the *ELF7-like* gene correlates negatively *CcFLC* during flowering (Figure 4). Both putative floral repressors *MAF1* and *MAF2* homologs transcribed synchronously during hickory flowering. GI is a light-dependent negative regulator of SPY in *A. thaliana*[42], as the homolog is the same with the results in hickory. In *A. thaliana*, direct interaction of AGL24 and SOC1 integrates flowering signals [43]. In hickory, both homologs work simultaneously except at S2.

#### Timing transcript abundance of putative flowering or floral genes in hickory compared with that in *A. thaliana*

**Figure 5:**
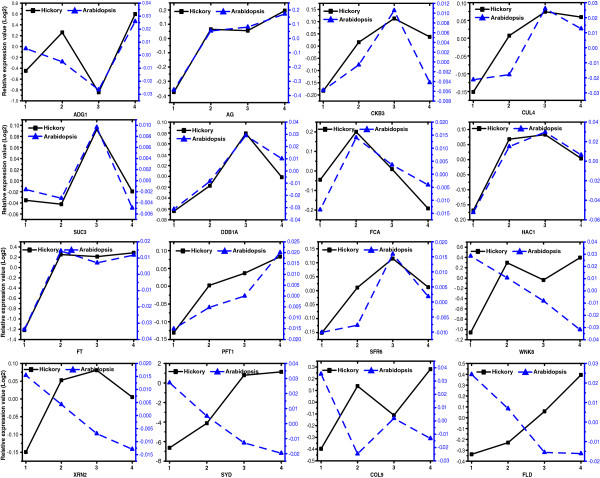
**Timing expression of flowering or floral genes in hickory compared with that in *A. thaliana*.** Flowering and floral developmental phase in hickory versus *A. thaliana* depending on morphological ontogeny. Phase 1: Stage 1–5 of the dataset from hickory and Stage 1–2 of the dataset from *A. thaliana* in which there is no morphological change. Phase 2: Stage 6 for hickory and Stage 3–4 for *A. thaliana* during which flower primordia of hickory and sepal primordia of *A. thaliana* are initiated. Phase 3: Stage 7 for hickory and Stage 5–6 for *A. thaliana,* in which pistillate flowers of hickory are developed and stamen of *A. thaliana* is initiated. Phase 4: Stage 8 for hickory and Stage 7 for *A. thaliana*, in which carpel development is initiated in both plants.

To obtain more insight into the hickory flowering mechanism, a comparison for flowering or floral core gene transcript abundance patterns was made between hickory and *A. thaliana* (Figure 5) [26]. Comparative microarray data of *A. thaliana* was downloaded from the website (http://www.ncbi.nlm.nih.gov/geo/query/acc.cgi?acc=GSE4594). Because of the different sampled time points of the two datasets from hickory and *A. thaliana*, the stages were firstly unified according to morphological comparison and totally four phases were captured. Phase 1 indicates Stage 1–5 of the dataset from hickory and Stage 1–2 of the dataset from *A. thaliana* in which there is no morphological change. Phase 2 indicates Stage 6 for hickory and Stage 3–4 for *A. thaliana* during which flower primordia of hickory and sepal primordia of *A. thaliana* are initiated. Phase 3 includes Stage 7 for hickory and Stage 5–6 for *A. thaliana,* in which pistillate flowers of hickory are developed and stamen of *A. thaliana* is initiated. Phase 4 denotes Stage 8 for hickory and Stage 7 for *A. thaliana*, in which carpel development is initiated in both plants [26].

Some flowering or floral genes or their homologs, e.g. *ADG1*, *AGAMOUS* (*AG*), *FT*, *HISTONE ACETYL TRANSFERASE OF THE CBP FAMILY 1* (*HAC1*), *CUL4*, *SUCROSE TRANSPORTER 3* (*AtSUC3*), *DAMAGED DNA BINDING PROTEIN 1* (*DDB1*), *CASEIN KINASE II BETA SUBUNIT 3* (*CKB3*), *PFT1*, *SENSITIVE TO FREEZING 3* (*SFR6*), *FCA*, are transcribed abundantly at the same way in both plants. For example, *FT*, a florigen in *A. thaliana*, and the homolog *CcFT* (GenBank: FJ858260.1) in hickory, express in a similar way in both plants. The expression of *FT* or the homolog shows an increase in the early stages and then maintains a high level through the later stages of floral development. *AG* specifies stamen and carpel identities. The homolog in hickory is designated as *CcAG* (GenBank: FJ858261.1). *AG* or *CcAG* shows a steady increase in transcript levels to promote pistillate flower initiation from Phase 2 in either *A. thaliana* or hickory. *FCA* or its homolog reaches a maximum in Phase 2 and decreases subsequently. PFT1 might act downstream of phyB to promote flowering in response to shade in *A. thaliana*[44]. While in the plant materials, *PFT1* or its homolog keeps transcribing during flowering in both plants.

However, some other transcripts are disparately abundant between in hickory and in *A. thaliana*. For example, in *A. thaliana*, expression of *CONSTANS-LIKE 9* (*COL9*) falls down sharply in Phase 2 and then maintains a low level in Phase 3 and 4. However, its homolog is opposite in hickory to that in *A. thaliana*. One possible reason is that the gene plays different roles in different plants. Another is that they are analogues but antagonize each other. Besides *COL9*, opposite patterns of other genes including *FLD*, *EXORIBONUCLEASE 2* (*XRN2*), *SPLAYED* (*SYD*), *SKB1*, *WNK8*, *INDETERMINATE-DOMAIN 2* (*IDD2*) or their homologs were identified based on transcript abundance comparison of those genes between *A. thaliana* and hickory.

In short, flowering or floral gene abundance in hickory is partly similar but partly particular to that in *A. thaliana*. Phase 2 (Day 1–3 after treatment in *A. thaliana*[26], and Stage 6 in hickory), is probably an important turning point from vegetative to generative phase in *A. thaliana* and hickory.

### Potential genes involved in hickory flower development

#### From co-expressed network

In order to identify potential novel genes associated with flower development, a co-expression network was constructed from a genome-wide co-expresser search for each flowering or floral core gene. The final network encompasses 295 nodes (genes) and 500 edges (co-expression interactions), corresponding to 232 contigs co-expressed with 62 putative flowering or floral core genes. GO enrichment analysis shows a significant enrichment of 42 GO terms for the co-expressed genes (Additional file 5: Table S4). A total of 27 contigs were strongly co-expressed with putative flowering or floral core genes (MR ≤ 30 and PCC ≥ 0.8) and involved in flower development based on GO annotation, which were selected as the potential genes involved in flower development (Additional file 6: Table S5). For instance, s1_contig16966 co-expresses with hypothetical flowering genes *CCR2* and *ELF4*. In hickory flowering, *ELF4-like* and *CCR2-like* show an opposite transcript abundance pattern (Additional file 7: Figure S1). The trajectory of s1_contig16966 transcript abundance is synchronous to *CCR2-like* but opposite to *ELF4-like*. It is inferred that s1_contig16966 possibly participates in photoperiod pathway and involves in photoperiodic perception and circadian regulation and alters *GI-like* expression to influence flowering time.

#### From function modules

**Figure 6:**
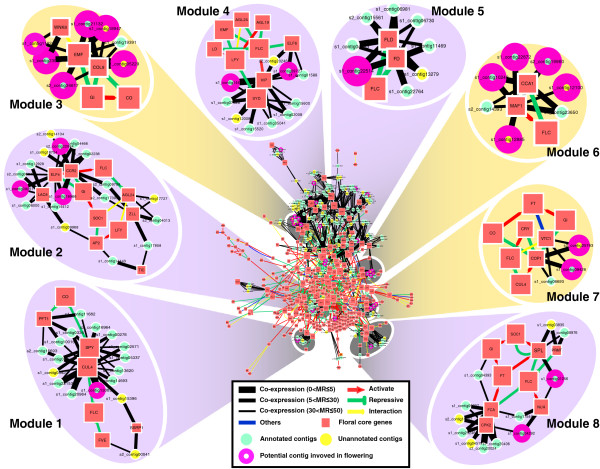
**Model of co-expression network with flowering function modules in hickory.** Model 1: Metabolic Process: Macromolecule metabolic process (36.36%), Cellular metabolic process(36.36%); Module 2: Mulitcellular organismal development (48%); Model 3: Development process: Reproductive development process (58.33%), Anatomical structure development (66.66%); Model 4: Regulation of biological process (64.29%); Module 5: Macromolecule metabolic process (60%); Model 6: Response to stress (44.44%); Module 7: Response to stimulus: Response to abiotic stimulus (80%), Response to stress (80%); Module 8: Cellular metabolic process (76.2%).

To discover unknown contigs as potential flowering or floral genes from the co-expression network, totally eight function modules was finally constructed including three modules (3, 6 and 7) directly associated with flower development (Figure 6).

Module 3 is enriched in the function of reproductive development process and the anatomical structure development including three unknown genes. Of them, two unannotated contigs (s1_contig18947 and s1_contig05229) strongly co-express with homologs of *EMF1* and *COL9* (Figure 6, Additional file 6: Table S5). It is suggested that the two contigs are possibly potential genes involving in flowering even keeping close relationship with homologs of *EMF1* and *COL9* in photoperiod pathway.

Module 6 which is enriched in “response to stress” contains three putative core flowering genes (*MAF1*, *CCA1* and *FLC* homologs). MAF1 represses flowering response to cold stress [45]. CCA1 also responds to coldness (GO: 0009409). In this module, there are seven contigs including two unknown contigs (s1_contig12100 and s1_contig12885) that co-express with the either one or both of homologs of core flowering genes.

Module 7 including 8 hypothetical core flowering genes (homologs of *COP1*, *VTC1*, *CRY*, *CUL4*, *GI*, *CO*, *FT* and *FLC*) is significantly enriched for the GO terms “response to abiotic stimulus” and “response to stress”. Three contigs (s1_contig09426, s1_contig06693 and s2_contig25763) co-express with the two homologs of core flowering genes of *COP1* and *VTC1*. The s1_contig09426 shows homology to plant serine/threonine-protein phosphatase 5 in plants. The s1_contig06693 shows sequence similarity of homogentisate geranylgeranyl transferase (HGGT). The s2_contig25763 is an unknown gene. These three contigs transcribe in same way as both core flowering genes do. It is predicted that these three contigs are possibly related to flowering in hickory.

Of the 21 potential genes captured from the co-expressed networks described above, total of 17 potential genes are assigned in the eight modules. Then, eight potential genes attribute in three flowering modules. Only four contigs have not been attributed to any modules (Figure 6, Additional file 6: Table S5). In addition, 6 unannotated contigs were recruited from flowering related modules which strongly co-expressed with homologs of well-known flowering or floral core genes into the potential gene group. These contigs are possibly new flowering or floral genes that are even unknown (Figure 6, Additional file 6: Table S5).

#### Validation of microarrays and the co-expression network for potential genes

To validate microarray measurements and further to verify the reliability of constructed hickory floral development gene network, total of 59 contigs including some identified potential floral genes and their co-expressed floral homologous genes were selected from the model of floral development gene network in hickory to perform quantitative-real time-reverse transcription PCR (Q-RT-PCR) which is one of the most robust and common approaches. The Q-RT-PCR verifications suggest that the microarrays can give the considerable results and the constructed co-expressed network is reliable (Additional file 7: Figure S1). For instance, s1_contig09426 is regarded as a potential gene captured from module 7, which co-expresses with one of hypothetical floral core genes *COP1*. The transcript abundance pattern of potential gene s1_contig09426 and *COP1-like* has a considerable fitness between Q-RT-PCR and microarray measurement (Additional file 7: Figure S1). The similar results with many other co-expressed gene pairs such as s2_contig22884 vs. *CCR2-like*, and s1_contig18947 vs. *COL9-like*, etc. are concluded (Additional file 7: Figure S1). The Q-RT-PCR verification further proved that the identified potential genes are reasonable and probable. These potential genes were regarded as candidate floral relative genes whose biological functions need to confirm in further research (Additional file 6: Table S5).

## Discussion

### A hickory flowering model

Flowering in hickory is triggered by several pathways synchronously including the photoperiod, autonomous, vernalization, gibberellin, and sucrose pathways (Figure 3). Recently, a new flowering pathway i.e. ambient-temperature pathway was mentioned [46,47]. Nevertheless, the genes involved in the ambient-temperature pathway such as *FVE, FCA, FLC, FT* and *SHORT VEGETATIVE PHASE* (*SVP*) and so on were also known as the genes in the five pathways. Current flowering network based on *A. thaliana* could response to the ambient-temperature influence, although the ambient-temperature pathway was not considered as an independent in the current flowering gene regulatory network. These environmental signals and internal cues from various pathways are possibly integrated by hypothetical floral integrators such as *CcFT*, *FD-like*, *CcLFY*, *CcAP1* (GenBank: EU155118, an *AP1* homolog in hickory) and subsequently initiate floral organ development.

Co-expression network in hickory includes eight function modules in which three are directly associated with flower development (Figure 6). In module 1, s1_contig13083 co-expresses with *SPY-like* and *CUL4-like* and involved in macromolecule metabolic and cellular metabolic processes based on GO annotation. This contig is an ubiquitin activating enzyme E1 (UBA1) via the blast result. A recent research shows that CUL4-DDB1 may function in the photoperiod pathway by interacting with SPA-COP1 complex [48]. It is possible that s1_contig13083 plays a role in the photoperiod pathway correlated with CUL4-like and SPY-like in hickory. In module 2, s1_contig16966 co-expresses with *CCR2-like* and *ELF4-like*. The contig is a homolog of glycosyltransferase in *A. thaliana* or *Populus trichocarpa* through NCBI blast. It has been identified that glycosyltransferase promotes flowering [49].

Moreover, s1_contig10248 co-expresses with *MAF1-like* and *CCA1-like*. In *A. thaliana*, MAF1 and CCA1 repress flowering response to cold stress [45]. It is inferred that s1_contig10248 is possibly a gene responding to coldness and functions in the vernalization pathway. The fact of s1_contig04635 co-expresses with *CcAP1* shows that the contig possibly plays a role in floral meristem identity and development in hickory. And, s1_contig22514, which co-expresses with *FD-like*, is a promising gene possibly functioning in floral signal integration in hickory.

Furthermore, 6 unannotated contigs co-express with corresponding flowering or floral genes respectively in flowering modules. Three of them, s1_contig11921, s1_contig05229, s1_contig18947 belong to module 3 whose GO function occurs in reproductive development process or in anatomical structure development. The s1_contig11921 co-expresses with *WNK8-like* and *EMF1-like*. It is speculated that s1_contig11921 might regulate flowering in photoperiod pathway together with *WNK8-like* and *EMF1-like* in hickory. The s1_contig18947 and s1_contig05229 co-express with flowering time genes *COL9-like* and *EMF1-like*. This shows that both contigs perhaps delay flowering by repressing *GI-like*, *CO-like*, or *FT-like* genes in hickory. The s1_contig12885 and s1_contig12100 are other two unannotated contigs in flowering module 6 which is involved in response to stress. Both contigs co-express with an *MAF1* relative. *MAF1*, similarly in amino-acid sequence to *FLC*, represses flowering response to cold stress [45]. All these suggest that both contigs play a role in cold stress in hickory as MAF1 does in *A. thaliana*. The last one unannotated contig s2_contig25763 belongs to module 7 which responds to abiotic stimulus and stress. It co-expresses with *COP1-like* and *VIC1-like*. *COP1*, a down-regulated gene of *PHYA*, is activated by *PHYA*. Various photoperiodic and autonomous flowering pathway mutants are epistatic to the *vtc1-1* mutant [50]. These results suggest that unannotated contig s2_contig25763 likely responds to stimuli or stress such as FR-light in hickory.

### Floral integrators in hickory flowering

Crosstalk among pathways by floral integrators such as FLC, FT, SOC1, LFY, AP1 might explain how the multiple signals affecting flowering are coordinated. However, there is currently no direct evidence to illustrate that these genes are with similar functions in trees. *FLC* is less influenced by external environmental transient change during flowering in *A. thaliana*[51], while transcript abundance of its homolog is dropping down slowly in hickory. *FT* is mainly influenced by a photoperiod and circadian clock [23]. The transcript abundance of *CcFT* is sensitive to subtle environmental change and accumulates continuously and maintains a high level consequently in hickory. FD is a FT downstream and forms a FD-FT complex to initiate transcription of floral specification genes (e.g. *LFY*, *AP1*) [52]. However in hickory, *FD-like* keeps transcribing and reaches a maximum at S5 and drops down subsequently. It is suggested that *FD-like* is regulated not only by *CcFT* but also directly by other genes such as *CcFLC1*, *TFL1*. *CcLFY* transcribes more strongly and reaches a maximum at S5 and reduces steeply later. It is speculated that *CcLFY* acts as a switch to flowering in hickory and is possibly regulated not only by the analogous FT-FD complex but also by several up-stream genes such as homologs of *FWA*, *GAMYB*, *GCR1*, *LD*, *PNF*, *PNY*, *SIN1*, *SPL*, *SYD*, *TFL1*, *AGL19*, *AGL24*, *AtMYB3*, *EMF*, etc. Similar to that in *A. thaliana*, as *CcLFY* level is accumulated to a critical value, the floral organ development is initiated [53]. Nevertheless, *CcAP1* keeps fluctuating in a narrow area possibly because it is crucial to initiate floral organ development but is not necessary to form sepal or petal for naked pistillate flower (Additional file 8: Figure S3).

### *FLC*-like gene-based vernalization system in hickory

FLC encodes a MADS domain protein that acts as a flowering repressor in *A. thaliana*[38]. It is also a key floral integrator in both the autonomous and vernalization pathways in flower development in *A. thaliana*[54]. In this study, two contigs (s1_contig20110 and s2_contig24845) with the same complete ORF obtained from different samples (SampleA and SampleB, respectively) have top blast hits with a FLC-like protein in *Pyrus pyrifolia* var. *culta*, and also with those in *Vitis vinifera*, *Coffea arabica*, *Citrus trifoliata*, *Vitis labrusca* × *Vitis vinifera*, *Citrus trifoliata*, and *Beta vulgaris*.

To validate whether the *FLC*-related gene (s1_contig20110 and s2_contig24845) exists in hickory or not, their primers was designed and the corresponding full-length CDS was cloned from a mixture of cDNAs of developing floral buds (Additional file 9: Figure S2). Then, the 3 terminal flanking sequence including polyA were cloned (Additional file 9: Figure S2). It is thus proven that the *FLC*-related gene can be transcribed during flowering in hickory. In addition, the timing transcript abundance pattern of the *FLC* homolog during flowering was characterized by real-time RT-PCR and *in situ* hybridization. It has been shown that transcript abundance of the *FLC* homolog steadily decreased during flowering, which is in accord with the microarray data (Additional file 9: Figure S2). Moreover, the *FLC* homolog was mainly transcribed in SAM, axillary bud primordia and procambia (Additional file 9: Figure S2). These results are all in conformance with those in *A. thaliana*[55,56]. Finally, the complete CDS and its predicted protein sequence were submitted to NCBI database and are designated as CcFLC1, a homolog of flowering locus C in hickory (accession number: AFM31223.1).

Interestingly, *FLC* homologs were also detected in such other species as *pyrus pyrifolia* var. *culta* (NCBI accession number: BAI99733.1), *Vitis vinifera* (accession number: ACZ26524.1), *Coffea arabica* (accession number: ADU56823.1), *Vitis labrusca* × *Vitis vinifera* (accession number: ABR68644.1, AEG19540.1) and *Populus simonii × Populus nigra* (accession number: JQ714386.1) except those of Brassica family. Reeves *et al*. [57] showed that BvFL1, an FLC homolog in sugar beet, function as a flowering repressor in transgenic *A. thaliana* and is down regulated in response to cold stress. In *Poncirus trifoliata*, *PtFLC* is regulated by alternative splicing and experiences seasonal fluctuation at transcriptional level, which might be an *FLC* candidate gene in *Poncirus trifoliate* (citrus) [58]. With RNAi interference and CHIP analysis, it has been shown that PtFLC functions as a flowering repressor in citrus. Chen (2008) [59] reported that over-expression of a poplar FLC-like MADS-box responds to low-temperature during vegetative bud dormancy. These results suggest possible existence of an *FLC*-like gene in hickory.

Besides the *FLC*-like gene, several other hypothetical flowering time genes were also involved in the vernalization pathway, such as homologs of *SIRTUIN* (*SRT*), *VIN3*, *HISTONE B2* (*H2B*), *CURLY LEAF* (*CLF*), *NUCLEAR PORE ANCHOR* (*NUA*), *PHOTOPERIOD-INDEPENDENT EARLY FLOWERING 1* (*PIE1*), *ACTIN-RELATED PROTEIN 4* (*ARP4*), *ARP6*, *ELF7*, *ELF8*, *HAC*, *UBIQUITIN CARRIER PROTEIN* (*UBC*), *VERNALIZATION INDEPEDENCE* (*VIP*), *MAF*, and *MBD9*, which are upstream genes of the *FLC* in *A. thaliana*. For example, VIN3 functions as a transient repressor of the FLC that involves histone deacetylation after affected by cold stress, and this VIN3-mediated process is required for the establishment of FLC silencing [60]. H2B deubiquitination is required for transcriptional activation of FLC and proper control of flowering in *A. thaliana*[61]. AtUBC1 and AtUBC2 play redundant roles and are involved in activation of FLC transcript, consequently resulting in repression of flowering [62]. *FLC* is regulated by these genes and subsequently regulates the transcript of its downstream genes, which lead to delayed or early flowering.

Furthermore, pistillate flower buds in hickory differentiate in late March while the leaves initiate in early April and stretch fully in mid-April. The observations that the pistillate flower initiates between dormancy break and leaf stretch demonstrate that temperature might be a main environmental factor in pistillate flower initiation. The nature of the pistillate flower bud differentiation provides a potential vernalization pathway.

### 'AC model’ for hickory pistillate flower development

In hickory flowering network, ABC model homologous genes *CcAP1* and *CcAG* (but lack of class A gene *AP2-like*, class B genes *AP3-like* and *PI-like*) are possibly activated by some floral integrators and consequently initiate floral organ development. As we know, *AP1 i*s an essential floral meristem identifying gene combined with a switch gene *LFY* and initiate floral organ development. On the other aspect, *AP1* and *AP2* both belong to class A genes and specify sepal identity [12]. However, hickory pistillate flower is naked without sepal, petal, or stamen but wrapped by bracts. It is inferred that the class A gene *CcAP1* is indispensable for floral organ ontogeny, and class C gene *CcAG* is essential for carpel initiation. Nevertheless, based on BLASTN searches for *A. thaliana* genes, homolog genes of class B genes (*AP3* and *PI*) could not be identified in hickory. One possible reason is that pistillate flower of hickory is lack of perianth and stamens, which indicate the dispensable of *AP3* and *PISTILLATA* (*PI*) genes. Hence, here we propose an 'AC model’ for hickory pistillate flower development.

### Comparison of flowering regulatory network in four species

The molecular basis for flowering was studied using an annual Long-day plant (LDP) *A. thaliana*[22,63,64], an annual Short-day plant (SDP) *Oryza sativa*[65,66], a perennial poplar tree [17,20] and a perennial hickory. The comparison of the flowering network across these four species may provide a better understanding of the regulatory pathways and molecular mechanisms regulating flowering.

Many major genes regulating flowering detected can be identical among all the four species by the common or homologs of the flowering genes (Additional file 10: Table S6). Several signal transduction, signal integration and floral organ development genes in this case have also been reported from the other three species [17,20,63-66]. In signal transduction stage, there are 48 hypothetical flowering or floral genes detected in hickory, including 12 SampleA specific genes, 19 SampleB specific genes and 21 common genes for SampleA and SampleB. While in signal integration stage and floral organ development stage, there are 17 hypothetical flowering or floral genes detected in hickory, including 2 SampleA specific genes, 8 SampleB specific genes and 7 common genes for SampleA and SampleB (Figure 3).

In the photoperiod pathway of hickory, light receptors such as *PHYA-like* perceive light, and several circadian clock genes (e.g. *EMF*, *TIMING OF CAB EXPRESSION 1* (*TOC1*), *CCA1*, *LATE ELONGATED HYPOCOTYL* (*LHY*), *ELF3*, *ELF4*, *CCR2*) respond to circadian rhythm. Subsequently, down-regulated genes, including DCAF1 complex (CUL and DDB1), DWD complex (CIBs, SPA, CRYs, COPs) are expressed and alter GI expression and impel a florigen gene *FT* transcription and translation [32]. In LDP *A. thaliana*, the regulatory module for photoperiodic flowering consists of GI-CO-FT signaling pathway, which is active only during LD. The GI up-regulates the expression of CO and in turn CO activates expression of FT [23,67,68]. However, in SDP *O. sativa sativa*, the regulatory pathway is composed of OsGI-Hd1-Hd3a, which is active only in SD [69,70]. In poplar, *FT* orthologue expression is influenced by *CO* orthologue and increased in LD, which may be involved in the juvenile to adult transition [24]. In the vernalization pathway, VIN3 functions as a transient repressor of FLC by cold stress in *A. thaliana*[60]. In hickory, *VIN3-like* correlates negatively *CcFLC* during flowering, and numerous relative floral genes involved in this pathway are identified. These suggest that VIN3-like perceives low temperature and transmits subsequently cold signal to downstream genes such as FRIGIDA-like (FRI-like) complex, PAF-like complex (ELFs-like; VIPs-like), SWR1C-like complex (PIE1-like; ARPs-like), MBD9-like, MAF-like and UBC-like which alter *CcFLC* transcript [55]. In *A. thaliana*, it is clearly illustrated that FRI suppresses flowering by increasing the levels of *FLC* mRNA [39]. FLC represses expression of SOC1, which prevents up-regulation of FD in the meristem. FLC also inhibits transcription of *FT* in the leaf [56]. In *Oryza sativa*, Komiya *et al*. [71] reported that OsMADS50 acts in leaves upstream of RFT1 and the *OsMADS50* mutation abolishes Ehd1 and RFT1 expression in leaves, causing a non-flowering phenotype during LD. In poplar, Bodt *et al*. [63] postulated that several FLC homologs regulate the seasonal time of flower initiation in adult trees and overexpression of PtFD1 induced extremely early flowering in poplar when plants were grown under LD photoperiods. In hickory, the transcript abundance of *FY*, *FLD*, *FPA*, *FVE*, *LSD1-LIKE* (*LDL*) and *MULTICOPY SUPRESSOR OF IRA* (*MSIs*) homologs is accumulated or decreased by age and nutrients in autonomous pathway [72]. They might further repress *CcFLC* transcript and initiate flowering. In gibberellin pathway, SPY-like could commit GA signal to CcFT or CcFLC integrators. In the sucrose pathway, ADG1-like, SUC-like related to sucrose synthesis may alter the integrators such as CcFT, AGL24-like or CcFLC transcript abundance to promote flowering. In *A. thaliana*, the autonomous pathway acts upon the expression of FLC. Several genes act additively to suppress the expression of FLC [39]. The GA pathway also actively promotes flowering in *A. thaliana*. Under SD conditions, GA4 up-regulates LFY [73] and SOC1 [64], leading to flowering. In *O. sativa*, Rao *et al*. [74] reported that RFL promotes flowering and RNAi suppression of *RFL* strongly delays flowering. However, in poplar, constitutive expression of PTLF does induce solitary flowers and PTLF was less effective for inducing early flowering [17,75].

In conclusion, the study has showed for the first time the gene regulation model for pistillate flower development in hickory via the joint-approach of RNA sequencing and microarray analysis. A total of 114 putative flowering or floral genes including 31 differentially transcribed ones were discovered in hickory and exhibited in the network. Although the genome-wide co-expression network for the putative flowering or floral genes was proposed, further physiological and biochemical research on the functions and the relationships of these putative flowering or floral genes might show their biological roles in the pistillate flower development in hickory.

## Conclusions

Transcription dynamics of pistillate flowering correlated genes and their involved major functions were characterized based on the k-means clustering and GO annotation analysis of differentially transcribed genes, which provides system-level insights into the pistillate flowering. A total of 114 putative flowering or floral genes including 31 differentially transcribed ones were identified in hickory, whose location, function and dynamic transcript abundance analysis based on the constructed flowering network of *A. thaliana* predicts that flowering event of pistillate flower bud in hickory is triggered by several pathways synchronously including the photoperiod, autonomous, vernalization, gibberellin, and sucrose pathway. Totally 27 newly potential flowering or floral genes were recruited from the genome-wide co-expression network functional module analysis. Moreover, the analysis provides a potential FLC-like gene based vernalization pathway and an 'AC’ model for pistillate flowering in hickory. This study provides an available framework for pistillate flower development in hickory, which is significant for insight into regulation of flowering and floral development of woody plants.

## Methods

### Plant material and experiment design

Terminal buds from short-pod-branches in a 15-year-old hickory tree were sampled in Lin’an (30˚N, 119˚W), China every 2 or 3 days from the beginning of March to the early April in 2009.

The floral developing process was tracked through morphological, anatomical, and ultrastructure observation, combined with molecular identification in order to grasp the floral process and the critical point from vegetative growth to reproductive growth. The morphological characteristics were photographed and the temperature in the field was recorded. Using paraffin section method, the buds were dissected longitudinally. And, using scanning electron microscopy (SEM), the ultrastructure of buds was studied. *CcLFY* was cloned and the temporal transcript abundance pattern was carried out to identify the critical point of floral initiation (Figure 1). As a result, March 18^th^ in 2009 is the critical point of pistillate flower bud differentiation. And, initiation of floral bud differentiation at molecular level is about 4 days earlier than that at morphological or anatomical level.

Based on previous results, 8 samples were chosen namely, S1-S8 (Figure 1), representing 5 different flower ontogeny stages. Samples S1, S2, S3 and S4 were obtained before March 18^th^ corresponding to the flower bud undifferentiated stage. Sample S5 on March 18^th^ represents the critical point of floral developmental transition at a molecular level. Sample S6 on March 22^th^ is a critical point of floral differentiation at a morphological level. S7 and S8 represent bract generation and carpel initiation, respectively.

The pistillate flower buds of S1-S8 were collected respectively. Each frozen sample was ground in a stainless stell blender, and then in a stainless steel grinder, to give a fine powder. Total RNA extraction was performed as described by Wang *et al*. [76]. Isolated RNA was quantitated using a Nanodrop spectrophotometer. The equal amounts of RNA of pistillate flower buds of S1-S5 were mixed as SampleA and those of S6-S8 were mixed as SampleB. The total RNAs of SampleA and SampleB were used to transcriptome sequencing, respectively. Sequenced reads were assembled to contigs which were able to search ones related to flowering via blast analysis. Then microarrays were designed, in which probes came from all of contigs. Five micrograms RNA was used for cDNA synthesis using oligo dT-primer and Superscript II Rnase-Reverse Transcriptase (Invitrogen) according to the manufacturer’s instructions. Microarrays were hybridized with cDNA from S1 to S8 stage, respectively. Using transcript abundance pattern cluster analysis, Gene Ontology analysis and pathway analysis, the map of flowering network in hickory was constructed.

### 454 Sequencing and data analysis

SampleA and SampleB were sequenced with Roche 454 transcriptome sequencing technology (Shanghai Biotechnology Co., Ltd.) respectively as follows: Preparation and sequencing of the 454 sequencing library was essentially performed. After filtering the adapter sequences and low quality sequences, the clean reads were assembled using CAP3 software at the default parameters (overlap 40 bp, identity 80%). For identifying the flowering or floral genes of hickory based on 454 contigs, local BLAST database was created with the *A. thaliana* cDNA library obtained from the TAIR10 database (http://www.arabidopsis.org). BLASTN searches for *A. thaliana* genes were performed, which was chosen because it had a best study in flower development among the plants and it belongs to the angiosperms, dicotyledonous class which is the same with hickory. Throughout this study, it was considered that the top BLAST hit for each contig with e value < 10e-5, identity percentage ≥ 80% and coverage percentage ≥ 50%, which were retrieved using a Perl script.

### Probe preparation and chip analysis

To characterize the transcriptional hallmarks and molecular mechanism of flower ontogeny, RNA transcript abundance profiles extracted from progressively flowering and floral development including eight samples and three developmental stages were analyzed. Probes were designed on the basis of assembled 454 contigs and 109 flowering or floral core genes of *A. thaliana* consulted from more than 1000 literatures. Labeled cRNA was prepared and hybridized to Alligent GeneChip according to the manufacturer’s guidelines.

Signal and transcript values of each gene were obtained. Genes with normalized signal values of 'A’ (absent) in all samples were discarded from further analysis. An arbitrarily fourfold change criterion among the eight samples was selected as the differentially transcribed genes modified with flower development. Normalization of gene transcript abundance values was performed by dividing each transcript abundance value by the mean transcript of this gene across all samples and then taken the logarithm with 2 as the base. The total of differentially transcribed genes was divided into nine clusters by a k-means algorithm with MultiExperiment Viewer (MeV) (version 4.6.2) and Pearson Correlation as the default distance metric for KMC in MeV software was used for similarity distance computing. Further the GO analyses of whole microarray probe sets were performed against AmiGO (http://amigo.geneontology.org/cgi-bin/amigo/go.cgi). Then the significant enrichment GO terms for each cluster were examined using hypergeometric test with P-value ≤ 0.01 based on the whole microarray probe sets GO analysis results.

### Construction of hickory flowering co-expression network

A total of 30,029 genes with at least one 'P’ signal value among the eight samples were used as the data sets to construct flowering and floral gene co-expression network. Instead of constructing a network based on the whole data sets, it was simply considered that the genes co-expressed with flowering or floral key genes as a more robust approach to survey the gene regulatory relationship over flower ontogeny, which made further efforts help us to detect validated genes involving flower development. To quantify the similarity of the gene transcript abundance profiles, Pearson’s correlation coefficients (PCC) of each gene pair, was calculated following the formulas of the online help page (http://atted.jp/help/coex_cal.shtml) and further transformed into Mutual Rank (MR) value with the method descripted (http://atted.jp/help/mr.shtml). The genes having the MR ≤ 50 with the flowering or floral genes were selected to generate the co-expression networks. Three width of edges are used to draw the networks, that is, bold edges (MR ≤ 5), normal edges (5 < MR ≤ 30) and thin edges (30 < MR ≤ 50).

### Transcript abundance pattern verification

Real-time RT-PCR was performed to validate the transcript abundance pattern of *CcLFY* and candidate co-expression genes. Five micrograms RNA was used for cDNA synthesis using oligo dT-primer and Superscript II Rnase-Reverse Transcriptase (Invitrogen) according to the manufacturer’s instructions. Amplification of cDNA was performed in the presence of gene-specific primers and the SYBR Green PCR master mix (Applied Biosystems, Foster City, CA, USA) in MicroAmp Optical 96-well reaction plates with optical covers using an ABI Prism 7500 Sequence Detector (Applied Biosystems). Each sample was analyzed in biological triplicate, using individual plants and treatments to test for reproducibility. The reaction conditions were 50°C for 2 min, 94°C for 10 min, and then 40 cycles of 94°C for 15 s and 60°C for 1 min. All cDNA samples were included in triplicate in all assays. Primers were designed using Primer express software (Applied Biosystems). Relative quantification of gene transcript abundance data was carried out with the 2^-ΔΔCT^ or comparative *C*T method [77], where the threshold cycle (*C*T) indicates the cycle number at which the amount of amplified transcript reaches a fixed threshold. Transcript levels were normalized with the *C*T values obtained for the internal standard hickory actin.

### Availability of supporting data

The raw data of RNA-seq and microarray analysis has been submitted to the website: http://www.cls.zju.edu.cn/binfo/hickory/.

## Competing interests

There are no potential competing interests related to this manuscript.

## Authors’ contributions

JQH, BSZ, YJH and ZJW conceived and designed the experiments, and contributed reagents/materials. YJH, LLL and BSZ wrote the manuscript. HYJ, FFC, WZJ, QXZ carried out the experiments. MC, LLL and YJH contributed to analysis the data. All authors read and approved the final manuscript.

## Supplementary Material

Additional file 1: Table S1 Summary of 454 sequencing, assembling and annotation. Click here for file

Additional file 2: Table S7 Blast result and corresponding sequence information based on assembled 454 contigs. Click here for file

Additional file 3: Table S3 Summary of flowering or floral genes in *A. thaliana* and Hickory. Click here for file

Additional file 4: Table S2 Regulatory relationship of flowering or floral genes in *A. thaliana*. Click here for file

Additional file 5: Table S4 Enrichment GO anotation of genes in co-expression network. Click here for file

Additional file 6: Table S5 Potential genes involved in hickory flower development. Click here for file

Additional file 7: Figure S1 Validation of microarray analysis and co-expression network. Click here for file

Additional file 8: Figure S3 Expression and regulation relationship of floral integrators in hickory. Click here for file

Additional file 9: Figure S2 Cloning and transcribing characteristics of complete *CcFLC* mRNA. Click here for file

Additional file 10: Table S6 Comparison of flowering regulatory network in four species. Click here for file
