# Supplementary material for: Use of transcriptome sequencing to understand the pistillate flowering in hickory (Carya cathayensis Sarg.)
Source: BMC Genomics. 2013 Oct 10;14:691. doi: 10.1186/1471-2164-14-691 (PMC3853572; doi:10.1186/1471-2164-14-691)
Supplement: Additional file 7: Figure S1 — Validation of microarray analysis and co-expression network. [file 1471-2164-14-691-S7.pdf]

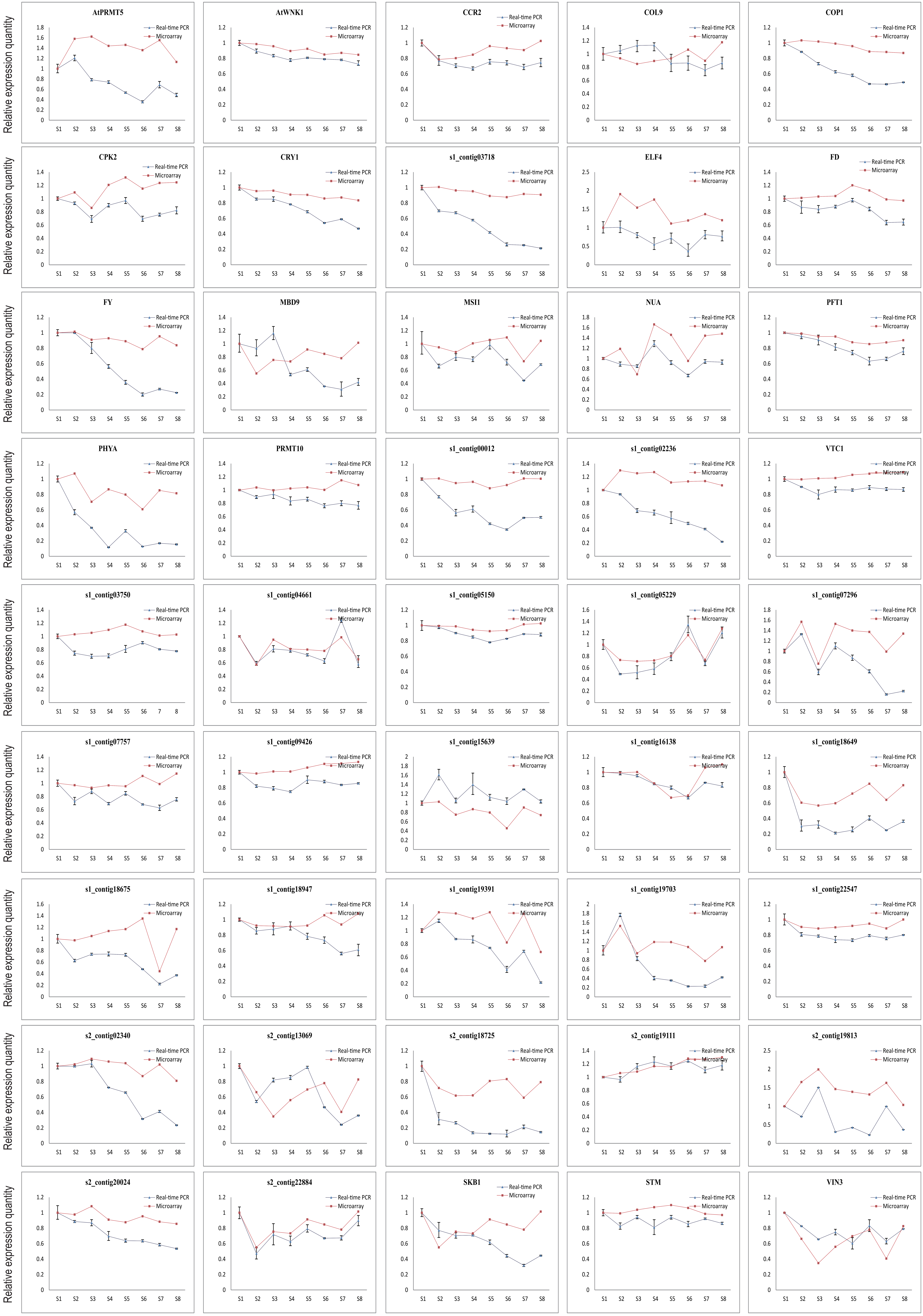
Relative expression quantity
Real-time PCR
Microarray
S1
S2
S3
S4
S5
S6
S7
S8
AtPRMT5
AtWNK1
CCR2
COL9
COP1
CPK2
CRY1
s1_contig03718
ELF4
FD
FY
MBD9
MSI1
NUA
PFT1
PHYA
PRMT10
s1_contig00012
s1_contig02236
VTC1
s1_contig03750
s1_contig04661
s1_contig05150
s1_contig05229
s1_contig07296
s1_contig07757
s1_contig09426
s1_contig15639
s1_contig16138
s1_contig18649
s1_contig18675
s1_contig18947
s1_contig19391
s1_contig19703
s1_contig22547
s2_contig02340
s2_contig13069
s2_contig18725
s2_contig19111
s2_contig19813
s2_contig20024
s2_contig22884
SKB1
STM
VIN3
