# Supplementary figures and images for: Use of transcriptome sequencing to understand the pistillate flowering in hickory (Carya cathayensis Sarg.)
Source: BMC Genomics. 2013 Oct 10;14:691. doi: 10.1186/1471-2164-14-691 (PMC3853572; doi:10.1186/1471-2164-14-691)

**a**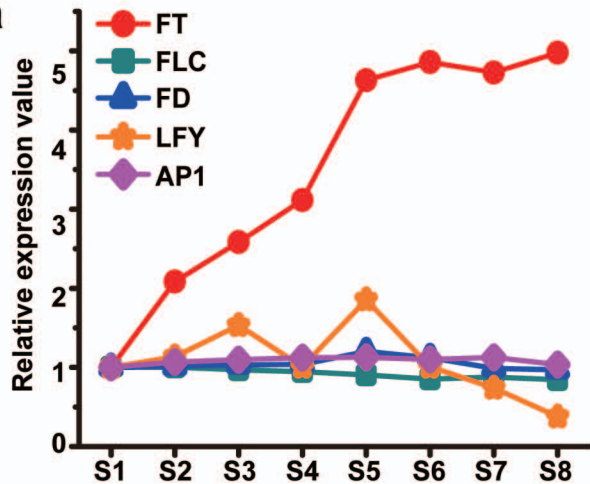**b**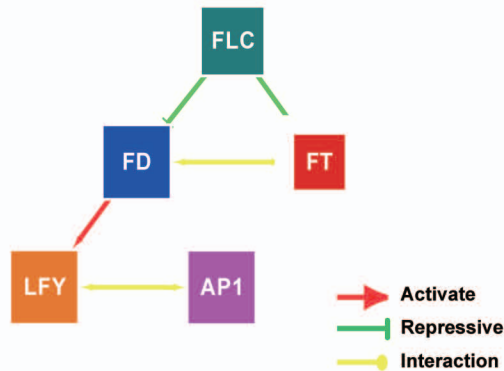

Supplement: Additional file 8: Figure S3 — Expression and regulation relationship of floral integrators in hickory. [file 1471-2164-14-691-S8.pdf]
