# Supplementary material for: Use of transcriptome sequencing to understand the pistillate flowering in hickory (Carya cathayensis Sarg.)
Source: BMC Genomics. 2013 Oct 10;14:691. doi: 10.1186/1471-2164-14-691 (PMC3853572; doi:10.1186/1471-2164-14-691)
Supplement: Additional file 9: Figure S2 — Cloning and transcribing characteristics of complete CcFLC mRNA. [file 1471-2164-14-691-S9.pdf]

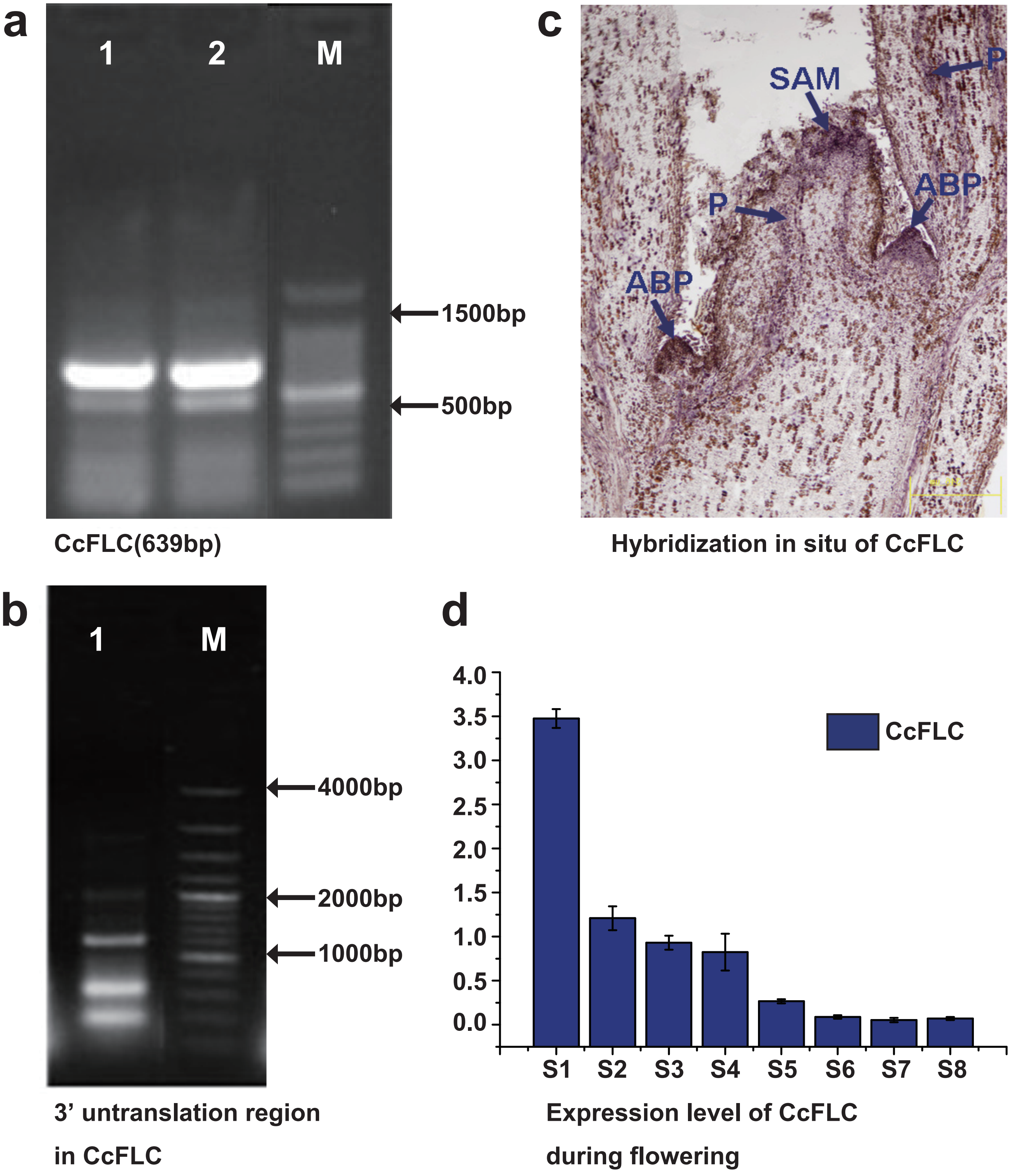

Figure S2 Cloning and expressing characteristics of complete CcFLC mRNA. a: Cloning of complete CDS sequence of CcFLC in hickory. b: Cloning of 3' untranslation region in CcFLC via 3'RACE analysis. c: Hybridization in situ of CcFLC homolog in the pistillate flower bud. SAM: shoot apical meristem; ABP: axillary bud primordium; P: procambia. d: CcFLC homolog expression level during flowering.
